# Aptamers: novelty tools for cancer biology

**DOI:** 10.18632/oncotarget.25260

**Published:** 2018-06-01

**Authors:** Ricardo L. Pereira, Isis C. Nascimento, Ana P. Santos, Isabella E.Y. Ogusuku, Claudiana Lameu, Günter Mayer, Henning Ulrich

**Affiliations:** ^1^ Department of Biochemistry, Institute of Chemistry, University of São Paulo, São Paulo, SP 05508-900, Brazil; ^2^ Chemical Biology and Chemical Genetics, Life and Medical Sciences (LIMES) Institute, University of Bonn, 53121, Bonn, Germany; ^3^ Center of Aptamer Research and Development (CARD), University of Bonn, 53121, Bonn, Germany

**Keywords:** aptamers, Cell-SELEX, clinical application, cancer

## Abstract

Although the term ‘cancer’ was still over two thousand years away of being coined, the first known cases of the disease date back to about 3000BC, in ancient Egypt. Five thousand years later, still lacking a cure, it has become one of the leading causes of death, killing over half a dozen million people yearly. So far, monoclonal antibodies are the most successful immune-therapy tools when it comes to fighting cancer. The number of clinical trials that use them has been increasing steadily during the past few years, especially since the Food and Drug Administration greenlit the use of the first immune-checkpoint blockade antibodies. However, albeit successful, this approach does come with the cost of auto-inflammatory toxicity. Taking this into account, the development of new therapeutic reagents with low toxicity becomes evident, particularly ones acting in tandem with the tools currently at our disposal. Ever since its discovery in the early nineties, aptamer technology has been used for a wide range of diagnostic and therapeutic applications. With similar properties to those of monoclonal antibodies, such as high-specificity of recognition and high-affinity binding, and the advantages of being developed using *in vitro* selection procedures, aptamers quickly became convenient building blocks for the generation of multifunctional constructs. In this review, we discuss the steps involved in the *in vitro* selection process that leads to functional aptamers - known as Systematic Evolution of Ligands by Exponential Enrichment - as well as the most recent applications of this technology in diagnostic and treatment of oncological illnesses. Moreover, we also suggest ways to improve such use.

## INTRODUCTION

Over the past few years, cancer has become one of the most important endemic health issues in the world [1, 2]. According to the World Health Organization, annual cancer cases are estimated to increase from 14 million in 2012 to more than 20 million by 2025 [3]. Not only due to its seriousness, but also due to the fact that it can have several different triggers - viral infection, environmental factors or genetic abnormalities – which lead to different levels of aggressiveness and/or progression [4]. Its level of complexity is paired only to that of its interest as a subject of study. The very same factors that may lead to its erratic behavior, challenge scientists throughout the world to come up with tools for histopathological evaluation in order to identify cancer cells among healthy ones, thanks to the different surface receptors that both cell types present. These receptors allow for the detection of cancer cells and its prognosis, as well as determining disease progression [5, 6].

Another important factor to consider is the proteome. Even though cancer has a strong link with the genome, the correlation of specific phenotypes to individual genotypes is key to leveraging the use of model organisms and patient samples in cancer research. In fact, cancer processes are mainly originated by damage or mutation of proto-oncogenes that code for proteins related to cell proliferation and differentiation, as well as the production of signals related to growth and apoptosis. Tumor development requires alterations in both oncogenes and tumor suppressor genes. These are favored by mutations in tumor susceptibility genes, which code for proteins related to DNA damage [7, 8]. This way, and because the proteome is much more dynamic, it is a more accurate source of information regarding what is happening inside the cell and therefore, a crucial tool regarding the disease itself and, of course, its progression. Furthermore, protein markers can be detected in body fluids and tissues, making them remarkable biomarkers, since their evaluation is noninvasive and, usually well-accepted by patients [9]. In the light of such concern, a novel therapeutic approach of immunotherapy has been developed in the past few years. Even though there is not enough long-term survival data available to make a strong statement regarding its efficiency, its practicality has made sufficient impact for one to realize that it has, indeed, redefined the standard-of-care treatment in the first-line settings [10].

Apart from surgery, the major therapeutic approaches for cancer are chemotherapy and biotherapy – the latter being a more biological approach, which includes immunotherapy and drug-delivery – and, given their versatility, aptamers can have an impact on both. Whether as stand-alone tools or in tandem with existing approaches, aptamers can be used both in treatment and diagnostics of oncologic illnesses with significant success.

Combinatorial chemistry is one of the most promising tools at the disposal of the pharmaceutical industry. In a glance, its use consists of three steps: synthesizing a new and random molecular library; selection against a specific target; and finally, characterizing the newly formed ligand-target complex. Nucleic acids are compounds of interest in this field, not only because they fold into well-defined secondary and tertiary structures, but also because they are rather easy to synthesize [11]. With that in mind, in 1990, Tuerk, Gold, Ellington and Szostak developed a technique that allows the isolation of nucleic acid molecules from a library with over 10^15^ sequences. This technique was then called Systematic Evolution [12]. SELEX (also referred to as *in vitro* selection or *in vitro* evolution) is a combinatorial chemistry technique used to produce oligonucleotides of either RNA or single-stranded DNA that bind to a particular target with high degrees of selectivity and affinity [13, 14]. Aptamers have been selected against a variety of targets including small molecules such as ATP [15] adenosine [16, 17], and proteins such as prions [18], as well as for inhibition of pathogenic vascular endothelial growth fator-induced (VEGF-induced) neovascularization [19].

Since the inception of this technique, both DNA and RNA aptamers have been selected against various targets – ions, small molecules or membrane receptors and even entire cells and organisms – and have demonstrated both affinity and specificity that pairs with that of monoclonal antibodies [20, 21].

Both monoclonal antibodies and combinatorial synthesized aptamers are being developed as site-specific drugs to bind to specific proteins that reveal altered activities in disease states. Antibodies have been considered promising therapeutic agents in various fields, especially cancer-related ones [22]. Aptamers have been conquering a solid reputation as an excellent alternative, given their high-specificity of recognition and high-affinity binding. Plus they have singular advantages such as *in vitro* development, the possibility of being developed against almost every target, no need for *in vivo* selection during their development, and the ability to be truncated to small biological-active sequences and modified for optimization [23, 24]. In addition to their discriminative recognition, aptamers offer advantages over antibodies as they can be engineered completely in a test tube, are readily produced by chemical synthesis, possess desirable storage properties and elicit little or no immunogenicity in therapeutic applications. All this makes them promising affinity ligands and distinct tools as affinity probes and analytical and therapeutic reagents [25–28].

Aptamers are usually created by selecting them from a large random sequence pool, however, it is possible to find them in their “natural form” in riboswitches.

Non-modified aptamers are cleared rapidly from the bloodstream, with a half-life of minutes to hours, mainly due to nuclease degradation and clearance from the body by the kidneys, a result of the aptamer's inherently low molecular weight [24]. This rapid clearance, alongside the already mentioned high selectivity and affinity, represent advantages in applications such as *in vivo* diagnostic imaging. Their high binding affinity and specificity, among other characteristics, has made them attractive diagnostic applications to target intra- and extracellular components of key signaling pathways. One such example is the already known RNA aptamer against the epidermal growth factor receptor (EGFR), which allows detection and/or determination of the extent of glioblastoma multiforme, as reviewd by Germer et al. [29]. In fact, their reputation has been growing immensely in the past few years, they are considered promising tools in the treatment of various pathologies [30], and some are actually undergoing pre-clinical trials [31].

## SELEX

Typically, the SELEX process is an iterative one; the selection of sequences that will bind to the target takes place after a few repetitions of the selection process, with increasing stringent conditions.

The starting point of the SELEX technique is the synthesis of a library of single-stranded DNA (ssDNA) containing 10^15^ different sequences, each one with a random region with 16 to 75 positions flanked by two constant regions, where primer annealing occurs.

The ssDNA is then folded and then the ssDNA is exposed to its target in order to select the sequences that constitute the desired aptamers. The ssDNA pool is incubated with its target and the best fitting molecules are collected and amplified using PCR procedures. Reiterative SELEX rounds are performed with increasing stringency to ensure the identification of the binders with the highest affinity [23].

It is, in its essence, an evolutionary process consisting in variation, selection, and replication [12], which can take up to 20 cycles to obtain the desired affinity [32].

**Figure 1:**
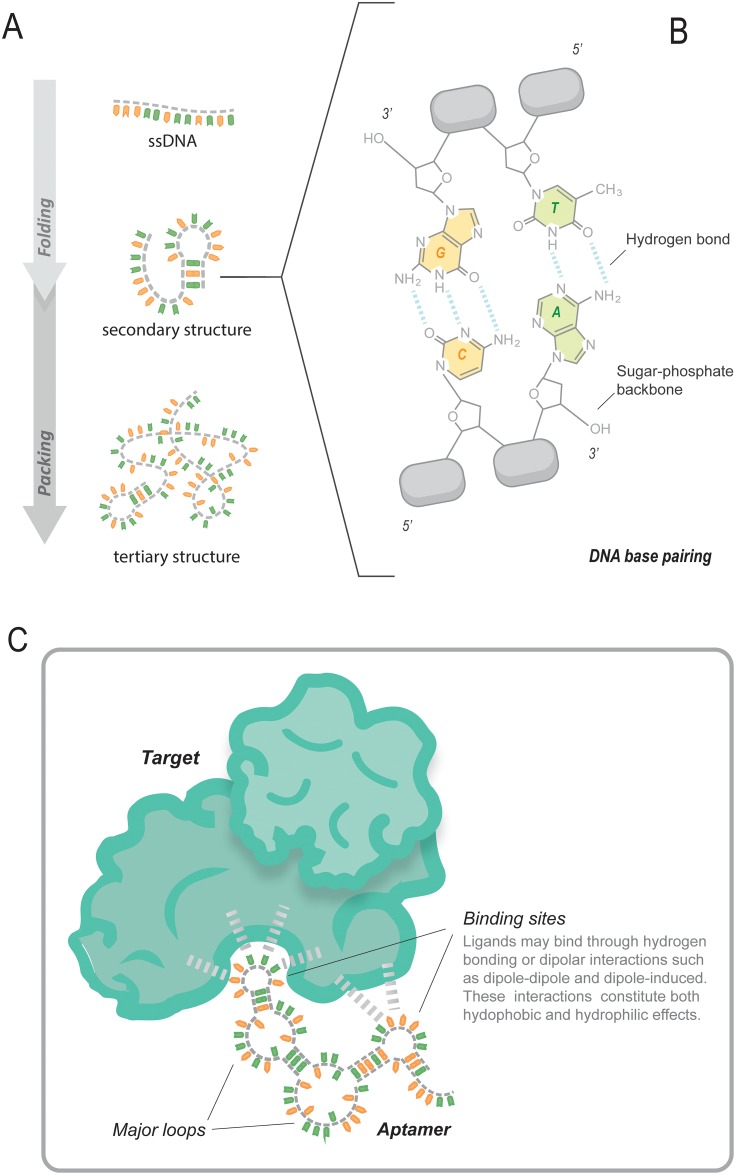
Depiction of the aptamer structure and its interaction with the target **(A)** The ssDNA is subjected to a set of conditions that enable it to fold and adopt a secondary structure. **(B)** The ssDNA base pairs in the linear parts of the molecule interact via hydrogen bonds, **(C)** in the loops, however, the bases are free to interact with the targets and do so via hydrogen bonds and/or dipolar interactions.

Based on their three-dimensional structure, aptamers can bind to a wide range of targets by Watson-Crick base-pairing, Hodgsteen-type or reverse type of base pairing, Van der Waals, hydrogen bonding, and electrostatic interactions [33, 34]. The specific loops are responsible for the interaction with the target (Figure 1).

In order to select the desired aptamers, the starting library of nucleic acids is incubated with the target molecule of interest. The nucleic acid ligands that adopt conformations allowing them to bind to the specific target are partitioned by filtration using either nitrocellulose (for protein target) or affinity chromatography (normally for small molecules target). The molecules that bind the target are eluted and amplified by RT-PCR (for RNA libraries) or PCR (for DNA libraries). This step yields a new pool that contains more target binding species.

**Figure 2:**
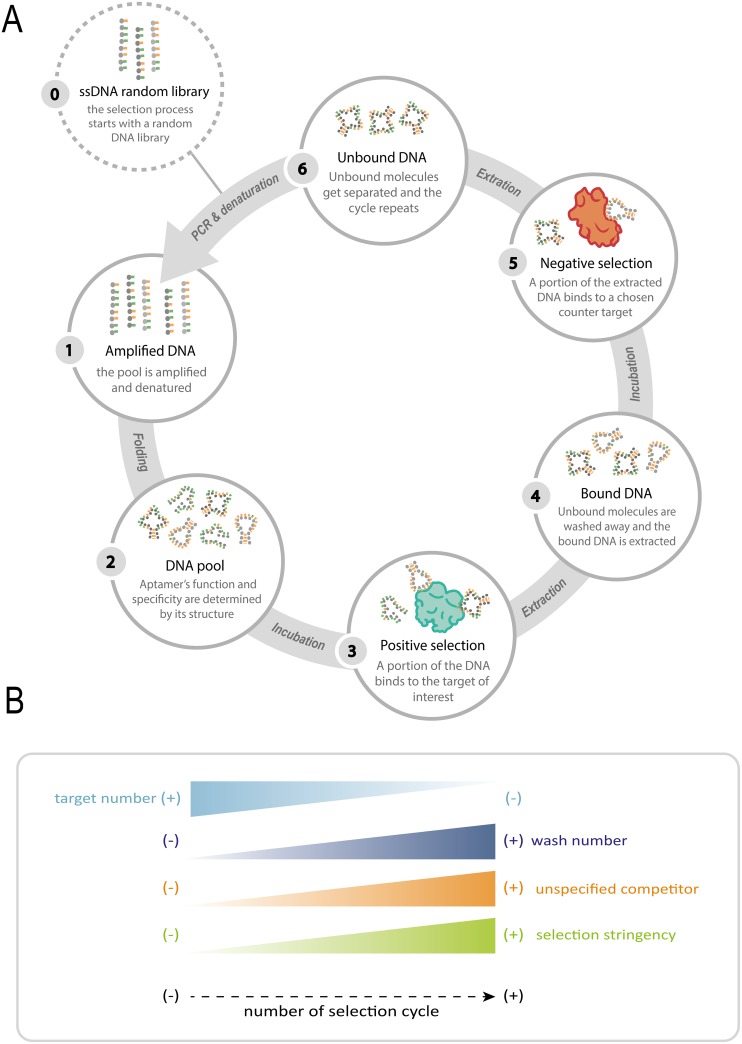
Schematic representation of the SELEX process **(A)** Not only is the process iterative, but also **(B)** with each round, the stringency is increased, thus resulting in higher affinity and specificity. This increase is achieved by increasing both the number of washes and non-specific competitors, and decreasing the number of target molecules within every cycle.

Being an iterative process, the methodology used to select the desired sequences has to be repeated several times (Figure 2A). The number of cycles required to obtain functional sequences depends on the stringency imposed to each round as well as on the affinity between the target and the aptamers (Figure 2B). Once the selection process is completed, an oligonucleotide population dominated by target-binding sequences is obtained. Cloning and sequencing of the selected clones will reveal the sequence and allow predicting the structure of the selected ligands.

## APTAMERS, CELL-SELEX AND CANCER CELLS

Early diagnosis and efficient eradication of tumor cells with minimum side effects on healthy cells are two of the top oncological challenges. Identification of biomarkers and targets exclusively (or differentially) expressed by tumor cells represent a significant contribution to determining prognosis, prediction of response to treatment, monitoring disease progression, development of new therapeutic and diagnostic strategies [35]. In an attempt to differentiate the molecular signature of tumor cells, several studies have used cell-SELEX technology to perform *in vitro* selection of aptamers against whole living cells. One of its advantages is that it does not require any previous knowledge regarding the target, thus eliminating the need to use the purified target during the procedure, which, in its natural environment, would yield aptamers against the native conformation of the target molecule.

The cell-SELEX procedure consists in the incubation of a target cell with random oligonucleotides (ssDNA or RNA), followed by the removal of the unbound aptamers and elution of aptamers bound to the cells, which are subsequently amplified and used in the next round of selection. This process is repeated several times until aptamers with high affinity and specificity are obtained. To increase their specificity, rounds of negative selection (also called counter or subtractive SELEX) are included. The pool of aptamers is incubated with cells morphologically, biologically and/or biochemically similar to the target. This can (and often is) also be done using the matrix where the (or the aptamer) is fixed [36]. To increase the efficiency of the process and to obtain aptamers with greater affinity for the target, it is advised to increase the stringency after the initial cycles. This can be attained by reducing the concentration of the target and increasing the concentration of the oligonucleotides in use, thus increasing the competition amongst the sequences. On the other hand, in negative cycles, it is suggested to use five to ten times more cells [37–40]. Some studies suggest that an automatic microfluidic system (on-chip cell-SELEX) can make the process faster and more efficient [41–43].

A variant of the cell-SELEX technique – the recently described isogenic cell-SELEX (iCell-SELEX) – proposes the use of genetic manipulation in order to enhance the positive and negative selection process. The overexpression of the target protein in the cells should, in theory, increase the effectiveness of positive selection rounds. On the other hand, using cells in negative selection rounds, whose expression of the target protein has been silenced, should increase their effectiveness [44]. This approach seems to be rather interesting regarding the selection of aptamer in the natural environment of the target protein, but can only be applied to known targets.

The cell-SELEX approach has generated several aptamers against various types of tumors including lung [45], glioblastoma [46], pancreas [47–49], colorectal [43] and liver cancer [50], lymphoma [51], leukemia and others [52–54]. Among other proposals for the use of aptamers in oncological diseases are the discoveries of new biomarkers for *in vivo* and *in vitro* imaging, diagnosis, and targeted drug delivery (Table 1).

**Table 1:** Aptamers selected using the cell-SELEX procedure

Aptamer	Cancer types	Target	Subtractive selection	Cycles of selection	Suggested function/application	Reference
GBI-10	Glioblastoma	U251 cells line		21	identification of targets	Daniels et al., 2003 [46]
-	Glioblastoma Multiforme	A-172 cell line	-	18	Diagnosis, targeted drug delivery, and discovery of molecular marker	Bayrac et al., 2011 [56]
GBM128 and GBM131	Glioblastoma	U118-MG cell line	SVGp12 cells	30	Identification of glioblastoma biomarkers	Kang et al., 2012 [57]
-	Glioblastoma	Tumor-initiating Cells (CD133^+^)	Non-stem glioma cells and neural progenitors cells	8	Aptamer-based therapies combined with conventional or targeted therapies	Kim et al., 2013 [58]
U2, U8, U19 and U31	Glioblastoma	U87 overexpressing EGFRvIII	U87MG cell line	11	molecular imaging probe	Wu et al., 2014 [59]
WQY-9-B	Gliosarcoma	K308	SVGp12	16	molecular probe for diagnosis	Wu et al., 2016 [60]
	Glioma	U87MG glioma cells	T98G cells	14	Discovery of new molecular targets	Cerchia et al., 2009 [61]
KMF2-1a	Breast cancer	MCF-10AT1	MCF-10A1		Cell type-specific intracellular delivery	Zhang et al., 2012 [62]
MS03	Breast cancer	MCF-7 cells	MCF-10A and MCF-7sal cells	13	Diagnostic and therapeutic applications	Lu et al., 2015 [63]
-	Breast cancer	TUBO cell line	CT26 cell line	12	Targeted breast cancer therapy	Moosavian et al., 2015 [64]
KW16-13	breast ductal carcinoma	MCF10CA1h	MCF10A	18	development as novel anti-tumor therapeutics	Chandrasekaran et al., 2016 [65]
TD05	B-cell lymphoma	Ramos cells	-	23	*In vivo* fluorescence imaging	Tang et al., 2007 [51]; Shi et al 2010., [66]
S3, S5, S12 and S27	Nasopharyngeal carcinoma	NPC 5-8F cell line	NP69 cell line	22	Identification of biomarker for early diagnosis and targeted therapy	Jia et al., 2016 [67]
-	Lung cancer	NCI-H69 SCLC cell line	NCIH661 NSCLC cell line	25	lung cancer subtyping during screening and appropriate treatment planning	Chen et al., 2008 [68]
	small cell lung cancer	SBC3	RERF-LC-MA	16	Specific detection probes	Kunii et al., 2011 [69]
TLS1, TLS3, TLS4, TLS6, TLS7, TLS9, and TLS11	Liver cancers	BNL 1ME A.7R.1 cell line	BNL CL.2 cell line	16	Development of molecular probe	Shangguan et al., 2008 [70]
	Liver cancer	HepG2	THLE-2	19	targeted therapies, and imaging probe	Xu et al., 2015 [50]
-	Hepatocarcinoma	HepG2 cells	Primary normal human liver hepatocyte cells,	11	Selective delivery of anticancer drugs	Ninomiya et al., 2013 [71]
LY-1, 13, 46, 32, 27/45, and 7/43	Hepatocellular carcinoma	HCCLM9 cell line	MHCC97L cell line	10	Identification of new diagnostic targets and developing new targeted therapeutics	Wang et al., 2013 [72]; Chen et al., 2016 [39]
LY-1	Liver cancers	HCCLM9	MHCC97L	9	Development of molecular probe and chemotherapy for metastatic hepatocellular carcinoma	Rong et al., 2016 [73]
-	Cholangiocarcinoma	QBC-939 cells	SMMC-7721	13	early diagnosis and therapeutics	Wan et al., 2015 [74]
-	Gastric carcinoma	AGS cell line	GES-1 cell line	12	Identify biomarkers for gastric cancer diagnosis and targeting therapy.	Cao et al., 2014 [75]
PL1-8	Pancreatic ductal adenocarcinoma	PL45 cell line	TOV-21G cell line	23	Biomarkers identification and drug delivery	Champanhac et al., 2015 [48]
XQ-2d	Pancreatic ductal adenocarcinoma	PL45 cell line	hTERT-HPNE cell line	15	*In vivo* imaging and clinical tissue recognition	Wu et al., 2015 [47]
Aptamers 1 and 146	Pancreatic cancer	HPAC cell line	HPDE cell line	16	CSCs targeting drug delivery, or circulating tumor cell detection	Kim et al., 2017 [49]
	Ovarian cancer	TOV-21G	HeLa	22	Identification of biomarkers	Van Simaeys et al., 2010 [76]
RLA01, RLA02, and RLA03	Ovarian cancer	Caov-3 cell	HOSE 6-3 cells	15	Diagnostic and drug delivery	Benedetto et al., 2015 [77]
-	Colorectal Cancer	DLD-1, Dukes’ type C colorectal adenocarcinoma	HCT 116	16	Identify specific biomarkers	Sefah et al., 2010 [78]
-	Metastatic colorectal cancer	LoVo cells	HCT-8 cells	22	multi-target cell imaging/ multi-target drug therapy	Li et al., 2014 [79]
-	Colorectal cancer	CR-CSC x HCT-8 CRC line	HCT-8 CRC line x CR-CSC	5	colorectal cancer cells and stem cells	Hung et al., 2015 [43]
XL-33-1	Colon cancer	SW620 cells	SW480 cells	14	metastatic cancer diagnosis and treatment	Li et al., 2015 [80]
-	Prostate cancer	PC3	HeLa and SMMC-7721 cells	17	Diagnosis and target therapy	Wang et al., 2014 [81]
-	Prostate adenocarcinoma	LNCaP cells	PC-3 cell line	10	Diagnostic and targeted drug delivery	Almasi et al., 2016 [82]
-	Prostate cancer	PC-3 cell line	RWPE-1	9	Identification of biomarker	Souza et al., 2016 [83]
-	Osteosarcoma	U-2 OS cell line	SGC7901, MCF-7 and HT-1080	13	specific diagnosis and developing probe-carrier-antitumor drug complexes and targeted therapies	Wang et al., 2015 [84]

Over the past few years, the number of cancer-related aptamers selected using cell-SELEX has increased significantly. Their applications are, clearly, diverse - from *in vivo* imaging to drug delivery, all the way to identification of new diagnostic targets.

In order to ensure that the findings of basic and translational research have an impact on clinical practice, it is not enough to select an aptamer or pool of aptamers against a tumor cell type. One must anticipate the desired applications for the resulting molecular probes, as this will be determinant for the definition of selection conditions, such as temperature of incubation with the target and/or the most appropriate counter targets. For instance, if the intent is to develop a diagnostic test that can identify circulating tumor cells, the aptamer should be able to differentiate a tumor cell from healthy blood cells. If an aptamer will be used as a tumor cell marker on a fixed tissue sample, it should not bind to healthy tissue. Moreover, problems arising from the selection conditions may interfere with process efficiency, such as contamination with dead cells in the positive selection cycle, which leads, to some degree, to the selection of aptamers against dead cells as opposed to the intended target [55]. Furthermore, the use of aptamers selected against live cells using fixed cells can lead to false positive results.

Some studies have successfully shown the use of aptamers for *in vivo* and *in vitro* imaging of tumor cells. Shi et al. [66], have shown, for the first time, the use of aptamer as a fluorescent probe for the identification of tumor cells (Ramos B-cell lymphoma) in living mice, proving the specificity, sensitivity and stability of aptamers for this purpose. More recently, the use of aptamers in the identification of pancreatic ductal adenocarcinoma tumor cells has been demonstrated by *in vivo* imaging and in fixed clinical specimens [47].

The identification of new biomarkers and, consequently, the development of specific aptamer-based biosensors, called aptasensors, which coupled with other techniques such as image or flow cytometry, microscopy and chromatography allow the detection and determination of the concentration of its target in the sample [24, 38, 85, 86]. Daniels and coworkers used cell-SELEX to select an aptamer- GBI10 –, which was shown to be a ligand for tenascin-C protein. It is interesting to note that, in this study, cell-SELEX cycles were performed at 4°C, to avoid receptor-mediated internalization [46]. However, if the aptamer does not bind well to its target at physiological temperature, it cannot be used *in vivo*. In view of that, the authors of the former cited work themselves suggested, for *in vivo* use, it would be necessary to develop a novel nuclease-resistant aptamer that binds the target with high affinity at 37°C. Similarly, Champanhac et al. performing selection cycles at 4°C, obtained aptamers with good affinity and specificity for their target, but some of these did not bind efficiently at 37°C. In addition, internalization of the aptamer was evaluated. For this, aptamers were incubated with the target at 4°C for 30 minutes, followed by incubation at 37°C for another 90 min. The internalization of aptamers by cells was assessed by treatment with trypsin leading to the exclusion of aptamers bound to the cell surface [48]. The authors suggested that these aptamers could be conjugated with drugs. However, it must be taken into account that the experimental design, which requires prior incubation at 4°C, does not reflect a proof of concept regarding the use of this approach in cancer therapy. It is noteworthy that low temperatures inhibit internalization of aptamers. Certainly, the ability of aptamers to be internalized by target cells makes them a promise for the development of therapies based on aptamers for intracellular drug delivery [87].

In addition to acting as a specific ligand, aptamers may also be used in the development of new drugs, as they can act preventing the activity of their target, for example, inhibiting a membrane receptor [88]. Selection of aptamers that may act specifically on tumor cells and may regulate the activity of molecules involved in key processes for tumor progression, such as proliferation, adhesion, migration and immune response is one of the greater goals in oncology-related aptamer research. Furthermore, it is important to determine the action of these aptamers inside and outside the cell, the toxicity in healthy and tumor cells, the existence of possible side effects and the half-life inside the organism.

## APTAMERS AS CHEMOTHERAPY AGENTS

Despite the most recent advances, cancer treatments, such as chemotherapy and radiotherapy, still have significant limitations regarding side effects, the major one being, of course, damage to healthy cells. Consequently, cancer treatments with minimal drug and radiation doses have been in the spotlight [89] due to the fact that they (attempt to) target, specifically, non-healthy cells. Some cell receptors expressed in normal cells are overexpressed in cancer cells; thus, efficiency could be enhanced whilst minimizing the damage to normal cells [90].

To improve the effectiveness of chemotherapy, some drugs have been conjugated with small molecules to decrease the resistance of cancer cells, such as Doxorubicin (DOX) – a drug used in many cancer treatments [91]. DOX is a drug approved by Food and Drug Administration (FDA) that acts as a genomic DNA intercalator, preventing DNA replication and is a member of the anthracycline family that may present some side effects, such as irreversible cardiomyopathy due to oxidative stress, downregulation of genes for contractile proteins, apoptosis and partial loss of myocardial fibers [92–95]. Subsequently, aptamer-based therapies are being studied in order to reduce such side effects. AS1411 is an antiproliferative DNA aptamer with a G-quadruplex structure developed to target nucleolin – a protein that is found in high levels in cancer cells. This oligonucleotide was the first aptamer that showed potential for clinical trial development in several cancer treatments and has been used in other studies to enhance the treatment efficiency against cancer cells [96, 97]. Li et al. developed an AS1411 aptamer system that releases DOX into human breast adenocarcinoma cells (MCF-7 cells). There was no evidence of drug accumulation in the heart of MCF-7 tumor-bearing mice. Binding of AS1411 to MCF-7 receptors was significantly increased when the micelle system was used. This led to the decrease of DOX accumulation in cardiac cells, thus reducing cardiac tissue damage and necrosis, providing a promising strategy for aptamer-based therapy [98]. Moreover, AS1411 reduced multi-drug resistance produced by glycoprotein-P (P-gP) efflux and showed antitumor activity and low levels of cardiotoxicity caused by DOX in MCF-7/ADR tumor-bearing mice [99]. This very same study used a bubble-generating agent that creates permeable defects in the bilipid layers when the microenvironment heats up to 42°C, which led to an increase of intracellular DOX concentration – a potentially harmful drug-releasing procedure. This limitation in therapeutic use of DOX could be overcome through the use of new aptamers binding to and inhibiting proteins necessary for P-gP production, leading to increased release of DOX and reduced viability of cancer cells without any cardiomyopathy side effects. Trinh et al. also used AS1411 and DOX in a hepatocellular carcinoma human model (HCC), showing no cardiac nor endocrine side effects, as well as no weight loss [100].

Atabi et al. showed aptamers that enhanced the storage of DOX in LNCaP cells (human prostate carcinoma cell line); Jing et al. constructed a double aptamer system that reduces human prostate cancer cell growth (LNCaP and PC3); Jeong et al. used mucin-1 aptamer (MUC-1) and DOX as treatment tools, on MCF-7 breast cancer cells and Sun et al. showed that the SL2B aptamer inhibits tumor growth in HT-29 cells (a human colorectal adenocarcinoma cell line) [101–104]. All these studies paved the way for the development for aptamers that can lead to lower-dosage chemotherapy, thus decreasing the aforementioned side effects. Aptamers binding to proteins or receptors involved in signaling pathways that lead to apoptosis and/or interfere with angiogenesis pathways are good examples of what could be done to improve such protocols. Another good example would be the use of an aptamer that inhibits topoisomerase II in order to reduce tumor cell proliferation.

Other types of drugs have been used in chemotherapy studies, some of which have been tested in conjugation with aptamers. Docetaxel (DTX) is an anticancer drug from the taxane family that causes tubulin depolymerization and microtubule aggregation, promoting cell death. However, it can cause intravenous toxicity in malignant brain tumors [105]. Gao et al. used AS1411 as an enhancer of the anti-glioma effect of DTX in C6 glioma cell line [106]. Glioma treatment is particularly difficult, due to the fact that both the blood-brain barrier (BBB) and the brain-glioma barrier reduce drug release from the blood into tumor cells. These types of studies can be improved with double aptamer systems – one for BBB recognition and another for tumor recognition.

Paclitaxel (PTX) is a neoplastic agent that acts as chemotherapeutic agent in malignant glioma cells [107], however, its lower aqueous solubility may affect its effectiveness. Aptamers could be selected to enhance the solubility of PTX to support the enhanced permeability and retention (EPR) effect of solid tumors. This application can increase tumor permeation and retention of macromolecular drugs to reduce toxicity to normal cells.

Taghavi et al. worked with anti-MUC-1 aptamer (5TR1) and enhanced Epirubicin (Epi) drug released into MCF-7 breast cancer cells and CHO cells (hamster ovary cell line) [108]. Epi is a drug of the anthraclycine family that can cause cardiotoxicity by accumulative doses. New aptamers could enhance recognition of MUC-1 glycoprotein to reduce cytotoxicity of myocardium cells improving Epi release to increase DNA intercalation, inhibit topoisomerase II and increasing cancer cell damage.

Ray et al. used an RNA aptamer to improve the effectiveness of Gemcitabine (Gem) drug chemotherapy targeting pancreatic tumor cells, which overexpress EGFR. This aptamer recognizes EGFR in pancreatic cancer cells and internalizes itself, along with Gem coupled to a polymer, resulting in inhibition of tumor cell proliferation with low toxicity levels [109]. Gem is known as a nucleoside molecule that can produce side effects in high concentration due the toxicity of normal cells, leading to neutropenia disease (low neutrophil concentration). To overcome limitations of this toxicity effect, aptamers that influence Gem phosphorylation status upon internalization could be used. Gem is a nucleoside that can be phosphorylated to allow inhibition of ribonucleotide reductase activity and subsequently preventing DNA elongation of proliferating cancer cells.

These studies show that there is much uncovered ground for aptamer-based chemotherapy. Whether by improving drug effectiveness or to reducing side effects (or both), aptamers can definitely improve cancer therapy.

## APTAMERS AS BIOTHERAPY AGENTS

### Aptamers and immunotherapy

Recent advances in cancer therapy have brought back to prominence the importance of the immune system in the tumor control. In fact, a major recent milestone in cancer immunotherapy has come with the unprecedented results obtained in clinical trials of advanced cancer patients treated with immune-checkpoint blockade antibodies. With such results, one cannot avoid wondering if the success of fighting cancer lies not only in the relationship between the drug and its target, but in an alliance between the drug and the human body. Here the drug may pave the way, and the body itself delivers the definitive blow. Combining different aptamer functionalities with other molecules of interest, such as reporter groups or proteins, provides a wide range of applications for aptamers as biotherapy tools. Aptamers have been regarded as rivals for antibodies for a long time, however, given the most recent findings, it makes sense to think that, maybe, both tools could work together. This is especially important when it comes to fighting cancer.

Zhao et al. developed a bivalent RNA aptamer, which inhibits the interaction of heat shock factor HSF1 with its cognate DNA promoter elements, thus down-regulating HSF1 expression and, therefore, sensitizing tumor cells to anti-cancer drugs [110]. In a different study, Lozano et al. developed an aptamer against Foxp3 by a CD28 2’-fluoro RNA aptamer [111]. The group has shown, as a proof of concept, that the P60 Foxp3 inhibitor peptide can be conjugated with a CD28 targeting aptamer for delivering the peptide to CD28-expressing cells, thus inhibiting T regulatory cells when they are hijacked by the tumor micro-environment to evade the immune response. Both works are very good examples of how regulatory proteins can be prevented from being an obstacle in therapy. Anticancer antibodies have certain limitations, when it comes to therapeutic applications, due to the ability of cancer cells to block immune responses. CD46 (membrane cofactor protein, MCP), CD55 (decay-accelerating factor, DAF)) and CD35 (complement receptor type-1, CR1) are well known for interfering with the aforementioned therapeutic agents. They protect normal tissues from accidental injury by activated complement, but also confer resistance to cancer cells, thereby limiting the effect of complement-fixing monoclonal antibodies. Designing aptamers that inhibit their cancer cell-protecting action would allow anticancer antibodies (or another aptamer) action. John Gordon Bruno has reported the use of two distinct DNA aptamers, developed by Ferreira et al., against MUC1 antigen [112]. In this study, aptamers were successful in enabling the death of MCF-7 cells when linked to the first component of complement (C1q) via a biotin-streptavidin system, resulting in the death of 50% of treated cells. Possible reasons for such effect include antigenic shedding *in vitro* and membrane-bound complement regulatory proteins (mCRPs) on the cell surface. CD46, CD55 and CD59 are good examples of such mCRPs. This is, yet, another case of a possible aptamer-immune system team-up for treating oncological diseases.

Immunotherapy is an effective and promising novel treatment strategy against several types of cancer, such as melanoma, non-small cell lung cancer, head cancer and neck cancer. Cytotoxic T lymphocyte protein-4 (CTLA-4) and programmed cell death protein-1 (PD-1) are critical immune checkpoint molecules that negatively regulate T cell activation, thus allowing tumors to escape the adaptive immune response. The use of immune checkpoint blockade-targeted antibodies has already shown clinical efficacy, which has led to their FDA approval in the treatment of several solid tumors [54, 113–115]. However, to this day, the medical community still lacks an accurate biomarker that allows appropriate patient selection [3, 116]. Aptamers could be used to identify such biomarkers and/or their variations in different cancer types. It is also important to note that treatment with such immunostimulatory therapies is not completely devoid of side effects, since cases of heart failure due to acute myocarditis have been reported [117, 118]. One of these particular studies, conducted by Läubli et al. revealed a correlation between the use of such drugs and impaired ventricular function [117]. Histological analysis of a myocardial biopsy revealed not only lymphocytic infiltration with a predominance of CD8-positive cells but, also, a reduction of FOXP3-positive regulatory T cells. Although symptomatic treatment is the only form of therapy for most forms of myocarditis [119], this particular scenario could benefit from the use of aptamers against these targets, since they could be used as biomarkers for the aforementioned elements and allow for an early diagnostic of this side-effect. The synergy between PD-1 and CTLA-4 has been proven to be very effective, and it is likely that this approach will continue to be used in the near future; however, while appealing, the double checkpoint inhibition strategy has a significant toxicity rate [114]. Aptamers could also play a significant role when planning combination therapies, in a sense that they could help identifying biomarkers involved in such adverse reactions. Malignant pleural mesothelioma (MPM), for instance, is a drug-resistant tumor arising from the mesothelial surfaces of the lung pleura, whose biomarkers lack specificity and sensitivity [120]. The development of accurate biomarkers for this disease could improve the treatment success rate significantly. CD44 is one of the most well-known MPM markers; however, it lacks an accurate measurement tool. Developing aptamers against this marker could help diagnosing this ailment sooner, which, in turn, would allow for a more planned course of treatment.

More recently, some groups have been turning their attention to another set of biomarkers, such as metabolic biomarkers. This set of markers is the most stable of all end-products among the four functional levels (genome, transcriptome, proteome and metabolome) related to cell function. Colo-rectal cancer (CRC) therapies have been taking advantage of this approach. Accumulation of lactate, phenylalanine, tyrosine, glutamine, proline, threonine, glutamic acid and arginine have, among a few others, have been identified as important CRC biomarkers [121–124]. Aptamers can be used to discover novel metabolic biomarkers, including the above-mentioned ones and especially those that are secreted by the cells. Using aptamers to quantify such metabolites can also prove to be advantageous for the study of cancer progression, since these metabolites can help understand the behavior of tumor cells and how they respond (if at all) to conventional therapies.

Ultimately, one day, it will be possible to predict which patients will benefit the most from a particular treatment, thus sparing them expensive and, sometimes, painful futile treatments. This idea has led research groups to study potential predictive biomarkers for personalized medicine, which span from gene expression signatures [125] to T-cell expression patterns within the tumor microenvironments. This approach would also benefit greatly from the aptamer technology, in a sense that aptamers could be used to analyze the presence of biomarkers, as well as variations in their concentrations, thus helping to design the best possible treatment.

### Aptamers as anticancer drug-delivery systems

Drug-delivery systems have been around for a while now, and they have, indeed, broken ground in terms of therapeutic approaches. Nanocarriers (colloidal nano-scale systems capable of transporting anticancer agents), such as small molecular weight drugs or macromolecules, for instance, do not only allow for treatment *in loco,* but they also protect the drug from degradation, reduce renal clearance, increase its half-life and help controlling release kinetics. Sometimes, they also improve solubility [126, 127]. However, the number of nanocarriers currently undergoing clinical trials is very small [128]. This can be attributed to the fact that, in general and especially in rapid-growth tumors, cancer cells are located adjacent to the endothelial barrier, and nanocarriers with targeting moieties will bind to the first receptors they find, ultimately failing to penetrate the rest of the tumor [128, 129]. This particular scenario is perfect for Cell-SELEX. Aptamers selected through this process bind to cellular receptors and may help the aforementioned nanocarriers by saturating the receptors that prevent them from penetrating the rest of the tumor.

**Figure 3:**
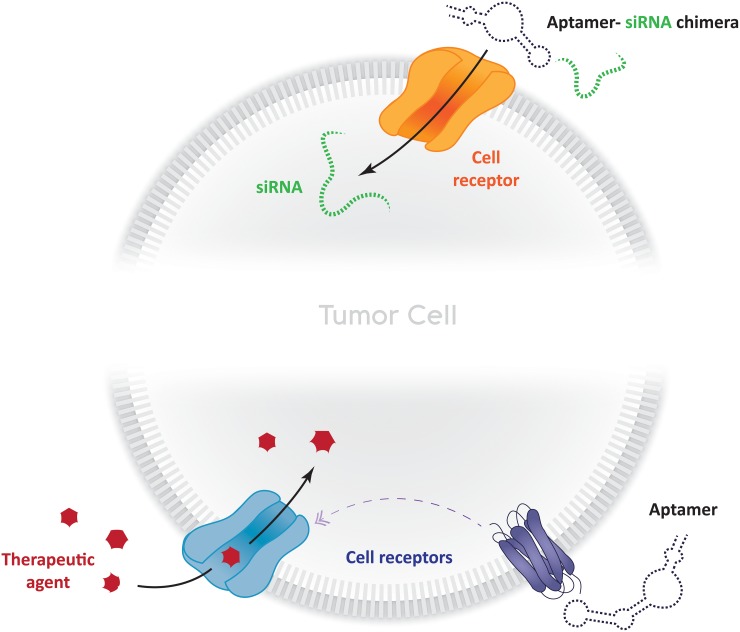
Strategies to enhance bio-therapeutic therapies in cancer using aptamers Aptamer-siRNA chimeras can be used to stimulate receptor internalization, which leads to siRNA delivery within the cytoplasm. Aptamers can also be used to induce cellular uptake of therapeutic agents (drugs, other aptamers, nanoparticles, polymers) which can act in different ways by apoptosis induction, lower RNA expression, tumor regression, and other mechanisms.

The use of RNA interference (RNAi), short interfering RNA (siRNA), targeting of micro RNAs (miRNAs) and antisense oligo (ASO) in cancer therapy for silencing gene expression has also been growing significantly [130]. However, even though most studies reveal that these tools are effective, their use is still filled with challenges that need to be overcome; mainly, the ones regarding their delivery, off-target effects and low concentrations in the target site. Aptamers can be used overcome these adversities. Receptor-mediated endocytosis, for instance, could account for concentration-related problems. Aptamers that target cellular uptake-related cell-surface receptors could be used to enforce this approach (Figure 3). Lipid-based drug delivery systems could also benefit from their use, given that one of their main drawbacks is the lack of control regarding their accumulation in tumor cells [131]. Berezhnoy et al. selected an aptamer (4-1BB aptamer-siRNA chimera against mTOR (Raptor)) that acts like this. Expression of mTOR (mechanistic target of rapamycin) reduces the differentiation of effector cells into T-memory cells. The 4-1BB aptamer targets activated T cells delivering the Raptor siRNA into the cell cytoplasm upon the internalization of 4-1BB (CD137); the siRNA within the cell inhibits mTOR favoring the induction of memory T cells [132]. Another good example of aptamer-siRNA chimeras that affect gene expression, is the one proposed by Dassie et al. The group managed to select a siRNA-binding aptamer that promotes regression of PSMA-expressing tumors [133] (Figure 3). This interesting idea could be expanded to, for example, metalloproteinases (MMP) and/or kallikrein (klk) secreting tumors. That very same group has also reported the use of an aptamer-siRNA chimera as a delivery agent. The group managed to select an RNA aptamer that delivers a cytotoxic siRNA to directly to prostate cancer cells for targeting prostate cancer-specific pro-survival genes, thus resulting in cell death [134, 135] (Figure 3). This was, in fact, the first study providing a proof-of-concept regarding the ability (and utility) that aptamers have to deliver functional RNAi to cells *in vitro* and *in vivo*.

In the aftermath of this revelation, other groups started to work in similar delivery systems. Liu et al. reported the use of an aptamer to deliver DOX to HER2-positive breast cancer cells. The aptamer-doxorubicin complex (Apt-DOX) was formulated by intercalating Dox into the DNA structure of HB5 and managed to selectively deliver DOX to HER2-positive breast cancer cells while reducing the drug intake by HER2-negative cells *in vitro* with reduced cytotoxicity to HER2-negative cells [136]. In a somewhat similar fashion, Thiel et al. reported the use of RNA aptamers as delivery agents of chemo-sensitizing siRNAs to HER2- positive breast cancer cells. The group used a novel, cell-based selection approach for isolating RNA aptamers that have cell-internalizing capabilities while being cell-type specific [137]. These aptamers were covalently linked to siRNAs targeting the Bcl-2 gene (anti-apoptotic gene). When applied to cells, the HER2 aptamer-Bcl-2 siRNA conjugates selectively internalized into HER2-positive cells and silenced Bcl-2 gene expression, thus sensitizing them to chemotherapy with cisplatin. Both works show tremendous potential, since the specific characteristics of cancer cells are what makes this ailment so singular and the nuances of its treatment so intricate. In the light of the fact that both act on the same type of cell via recognition of expressed proteins, it would be interesting to see how these aptamers would interact when “stacked” –using one aptamer that acts as an apoptotic inducer by targeting anti-apoptotic genes along with another that delivers an apoptosis inducer might prove even more effective than using only one of them.

More recently, AlShamaileh et al. reported showed that EpCAM-aptamer-guided surviving RNAi effectively downregulated survivin expression both in colorectal cancer cells *in vitro* and in a mouse xenograft model for colorectal cancer. The group identified survivin as one of the key players responsible for the innate chemoresistance of colorectal cancer stem cells and their aptamer-guided survivin RNAi enhanced sensitivity towards 5-FU or oxaliplatin in colorectal cancer stem cells, increased apoptosis, inhibited tumor growth and improved the overall survival of mice bearing xenograft colorectal cancer [138].

Whether they carry siRNAs or other specific therapeutic tools, such as drugs, aptamers can be used as carriers in target-based therapies. Following this line of thought, Bahreyni et al. developed a nanocomplex made of graphene oxide and aptamers for treatment of cancer cells. The group designed two nano-complexes (MUC1 aptamer-NAS-24 aptamer-Graphene oxide and MUC1 aptamer-Cytochrome C aptamer-GO) that induce cell death in two cancer cell lines (MDA-MB-231 and MCF-7) [139]. One of the aptamers (MUC1) acts as a probe promoting the internalization of the two nano-complexes. In turn, NAS-24 aptamer induces apoptosis via binding to vimentin expressed in cancer cells [37]. The cytochrome C targeting aptamer confirmed apoptosis induction promoted by the NAS-24 aptamer, since cytochrome C is known to have a significant role in the activation of caspase family which, in turn, triggers execution of programed cell death [140–142]. The complexity of this work reflects fairly well the infinite number of possibilities that aptamers (can) provide in terms of both therapeutic and diagnostic design in cancer biology.

## CLINICAL TRIALS

Despite the increasing number of published studies demonstrating advances in the development of aptamers applied to oncological diseases, the progress in the utilization of this knowledge in clinical practice has been very slow. Particularly with regard to the use of aptamers in clinical trials, the public clinical trials database (http://clinicaltrials.gov) lists around 33 clinical trials using aptamers for a wide range of therapeutic applications, of which only 5 are studies applied to oncology (Table 2).

**Table 2:** Clinical trials undertaken with aptamers targeting cancer

Trial number	Cancer type	Study phase	Primary purpose
NCT02957370	Bladder	Observational	Discover biomarker
NCT03385148	Colorectal	Phase 1	Diagnostic
NCT01830244	Breast	Phase 2	Treatment^*^
NCT00056199	Retinal	Phase 1	Treatment
NCT01034410	Acute myeloid leukemia	Phase 2	Treatment

^*^The aptamer is not the focus of the study. Samples of patients who agree will be used to isolate cancer initiating cells generate breast cancer models and using aptamers to target tumors.

The number of known cancer-targeting aptamers in clinical trials corresponds to, approximately, 5% of the total number of aptamers known to be in that development stage. This content is of public domain (https://clinicaltrials.gov).

As discussed throughout this review aptamers have several applications in diagnosis, prognosis determination, adjuvant therapy and targeted therapy. Subsequently, several research groups have been engaged in the development of aptamers whose targets are relevant for oncology, including proteins such as EGFR, HER-2, VEGF, EpCAM and others [34, 143]. Furthermore many of these researches result in patents, as can be seen in the WIPO (World Intellectual Property Organization) which lists hundreds of published international patent applications in this field of knowledge. These data make us reflect on a possible deficit in translational research that facilitates the arrival of knowledge generated in basic research to the clinic in an agile and efficient way.

## CONCLUSIONS

In the light of these examples, aptamers are definitely compounds of interest when it comes to cancer biology. Their applicability is rather vast, easy and has a low production cost. Also, considering how much ground there is to pave regarding cancer therapy and how versatile aptamers can be, their position as novelty tools is indisputable, whether as therapeutic and/or diagnostic tools or as complementary tools to already existing approaches.
